# Differences in Tolerance to Hypoxia: Physiological, Biochemical, and Molecular-Biological Characteristics

**DOI:** 10.3390/biomedicines8100428

**Published:** 2020-10-18

**Authors:** Dzhuliia Dzhalilova, Olga Makarova

**Affiliations:** Department of Immunomorphology of Inflammation, Federal State Budgetary Institution ‘Research Institute of Human Morphology’, Moscow 117418, Russia; makarov.olga2013@yandex.ru

**Keywords:** tolerance to hypoxia, high altitude, biomarkers, hypoxia-inducible factor, acute mountain sickness, high-altitude pulmonary edema

## Abstract

Hypoxia plays an important role in the development of many infectious, inflammatory, and tumor diseases. The predisposition to such disorders is mostly provided by differences in basic tolerance to oxygen deficiency, which we discuss in this review. Except the direct exposure of different-severity hypoxia in decompression chambers or in highland conditions, there are no alternative methods for determining organism tolerance. Due to the variability of the detection methods, differences in many parameters between tolerant and susceptible organisms are still not well-characterized, but some of them can serve as biomarkers of susceptibility to hypoxia. At the moment, several potential biomarkers in conditions after hypoxic exposure have been identified both in experimental animals and humans. The main potential biomarkers are Hypoxia-Inducible Factor (HIF)-1, Heat-Shock Protein 70 (HSP70), and NO. Due to the different mechanisms of various high-altitude diseases, biomarkers may not be highly specific and universal. Therefore, it is extremely important to conduct research on hypoxia susceptibility biomarkers. Moreover, it is important to develop a method for the evaluation of organisms’ basic hypoxia tolerance without the necessity of any oxygen deficiency exposure. This can contribute to new personalized medicine approaches’ development for diagnostics and the treatment of inflammatory and tumor diseases, taking into account hypoxia tolerance differences.

## 1. Introduction

Oxygen deficiency is one of the key factors in the development of infectious, inflammatory, and tumor diseases [1,2,3,4,5,6]. It is known that basic tolerance to hypoxia in humans and various animal species differs [7,8,9,10,11,12,13,14,15,16,17,18,19,20,21,22]. The differences in basic tolerance to oxygen deficiency can determine the predisposition to the development of hypoxia-related disorders. The evidence of this fact can serve, for instance, the variations in angiogenic responses to hypoxia, which may alter adaptive changes in coronary artery disease [23]. However, at present, the physiological, biochemical, and molecular-biological characteristics of hypoxia-tolerant and -susceptible organisms remain not well-studied. The research in this field could result in discovering the possible biomarkers of hypoxia susceptibility.

Animals are divided into tolerant- and susceptible-to-hypoxia groups in a decompression chamber at extreme altitudes. Humans are divided into tolerant and susceptible according to the acute mountain sickness (AMS) and high-altitude pulmonary edema (HAPE) sensitivity. Existing methods for determining resistance to a lack of oxygen, such as exposure in a decompression chamber, providing the conditions of the mountain environment, or breathing a hypoxic gas mixture, differ significantly and require standardization. In addition, in scientific reports of hypoxia tolerance investigations, species, age, sex differences and the influence of external factors—in particular, biorhythms—are not taken into account. Therefore, the data on tolerant- and susceptible-to-hypoxia humans and animals are difficult to compare. The mechanisms that determine the differences in resistance to oxygen deficiency also remain not fully understood.

In this review we discuss the physiological, biochemical, and biomolecular characteristics of humans and animals with different tolerances to hypoxia, taking into consideration various methods of their evaluation, as well as possible biomarkers for determining the basic resistance to oxygen deficiency without hypoxic exposure. Finding out the factors that makes individuals less susceptible to hypoxia, and diseases connected with such a condition, may benefit the treatment of patients with predispositions to these states. Furthermore, the study of differences between hypoxia-tolerant and hypoxia-susceptible organisms is also relevant for flight and space medicine since oxygen deficiency is a significant risk factor in space flights and extravehicular activities.

## 2. Cellular Response to Hypoxic Exposure

### 2.1. Structure and Functions of Hypoxia-Inducible Factor

A stable and continuous tissue oxygen supply is essential for the functional integrity of cells and the survival of mammals, including humans. Hypoxia accompanies the development of many diseases, including inflammatory, infectious, and tumor diseases. To provide the essential oxygen delivery to all cells, organisms have developed a complex physiological system over million years of evolution. The key factor providing a cellular response to hypoxia is Hypoxia-Inducible Factor (HIF)—the Nobel Prize (2019) was awarded for the discovery of this. It regulates the expression of several thousand genes—in particular, glycolytic enzymes, vascular endothelial growth factor (VEGF), and erythropoietin, etc. [24,25,26,27,28]. HIF is a heterodimeric complex consisting of one of the oxygen-regulated α-subunit isoforms (HIF-1α, HIF-2α or HIF-3α) and the constitutively expressed subunit HIF-1β [26,27,29]. Under normoxia, de novo synthesized cytoplasmic HIF-α is regulated by hydroxylation. This process is performed on proline residues by prolyl hydroxylases (Prolyl Hydroxylase Domain proteins—PHD1, PHD2, and PHD3) and on asparagin residues via the factor-inhibiting HIF (FIH) [30,31]. Two proline residues of HIF-α subunits are hydroxylated by PHDs. This triggers their identification by the E3 ubiquitin ligase complex, formed by von Hippel–Lindau tumor suppressor protein (pVHL), Cullin 2, elongin B and C, and RING-box 1 proteins. The hydroxylation happens thanks to PHDs reliant on oxygen, α-ketoglutarate, iron (Fe^2+^), and vitamin C [32,33,34]. Despite the fact that all PHDs are expressed ubiquitously, they demonstrate tissue-specific differences in expression levels. PHD2, the most predominantly expressed enzyme, could be detected in almost every tissue. At the same time, in the testis and heart, PHD1 and PHD3 are the most abundantly expressed enzymes, respectively. As for the context of HIF signaling, PHD2 plays the key role of an oxygen sensor [33,35]. Asparagine residue hydroxylation, performed by FIH, prevents the binding of the transcriptional co-activator histone acetyltransferase p300/CREB-binding protein (p300/CBP), therefore reducing the activity of HIF carboxy-terminal transactivation domain [33,34]. There are three major PHD isoforms, but there is only one known FIH isoform. Without FIH no hydroxylation of the Asn803 residue happens [31,36,37]. FIH and PHDs share some enzymatic properties. This fact suggested the hypothesis that PHDs and FIH are functionally redundant in their regulation of HIF. In addition, FIH has a lower Km for oxygen than the PHD enzymes [38,39,40]. It was demonstrated that the HIF-1α isoform is more susceptible to FIH modification than HIF-2α [41,42]. HIF-1α is the isoform directly regulating the expression of many enzymes, controlling the metabolism [43]; the differential control of HIF-1α by FIH could apparently trigger specific changes in metabolic response.

Under hypoxic conditions, HIF-α hydroxylation is weakened, it accumulates in the cytoplasm and translocates into the nucleus, where it dimerizes with the HIF-β subunit. The HIF-α/β dimer binds to hypoxia-responsive Hypoxia-Response Elements (HREs), which are located in the promoters of oxygen-dependent genes, involved in the systemic and cellular adaptation to hypoxia—glucose transporter genes, glycolytic enzymes, angiogenic and hematopoietic growth factors, etc. [44,45,46,47]. Despite HIF-1α and HIF-2α sharing very similar characteristics, including their potential to heterodimerize with HIF-1β, bind to HREs in hypoxia-inducible genes and their role in transcriptional activation, they differ according to the expression level during different developmental stages in various tissues [48,49,50]. HIF-2α is expressed most fully during the embryonic development stage and in vascular endothelial cells, placenta, lungs, and heart. At the same time, HIF-1α demonstrates ubiquitous expression in all analyzed cell types and mammalian tissues, especially in heart and kidney [48,51,52]. HIF-1α and HIF-2α display not identical specificity on their transcriptional targets. For example, HIF-1α effectively stimulates the expression of some proangiogenic genes, glycolytic enzymes, such as lactate dehydrogenase A, and pH regulation-involved genes. In comparison, HIF-2α affects more pronouncedly on EPO gene, matrix metalloproteinases genes, and iron metabolism-involved genes, while another group of genes, including VEGF and GLUT-1, is regulated both by HIF-1α and HIF-2α [43,53]. HIF-1 controls the acute adaptation to hypoxia, whereas HIF-2 and HIF-3 activity starts later, during chronic hypoxia, which provides a transitional switch between HIF proteins [54]. When HIF-1 levels become lower, HIF-2 and HIF-3 increase. Such a shift from HIF-1 to HIF-2 and HIF-3 signaling is important in order to provide the endothelium adaptation to prolonged hypoxia. The lack of ability to reduce the HIF-1 levels during prolonged hypoxia results in cell death [55]. It was revealed that the switch from HIF-1 to HIF-2 forms a universal mechanism of cellular adaptation to hypoxia and that HIF-1α and HIF-2α mRNA stability differences contribute to the HIF switch [56]. It is suggested that microRNAs are involved in the regulation of the content of different HIF isoforms. During prolonged hypoxia, microRNA expression varies to provide low HIF-1 functioning and maintain elevated HIF-2 and HIF-3 levels. Therefore, microRNAs have the ability to control the hypoxic HIF switch in human endothelium [55,57,58].

Except hypoxia, there are many other mechanisms of HIF activity regulation [26]. It was demonstrated that the degradation of HIF-1α is regulated in an oxygen-independent pathway, involving heat-shock protein 90 (HSP90) and receptor for activated C-kinase 1 (RACK1) [59]. Studies have shown that Transforming Growth Factor-β (TGF-β) is also closely related to HIF-1α: it inhibits PHD2, which, in its turn, activates HIF-1α [60]. Thus, some growth factors can control the stability of HIF-1α through the regulation of PHD2 and, therefore, trigger the expression of specific genes [60]. Furthermore, HIF-1α is activated in response to reactive oxygen species (ROS), inflammatory cytokines such as IL-1β, nuclear transcription factor NF-κB, the Phosphoinositide 3-Kinase (PI3K) signaling pathway and Mammalian Target of Rapamycin (mTOR) [26,28,61,62,63,64,65,66,67].

In addition, it was detected that the Kruppel-like factor 2 (KLF2), which is essential for normal vessel development, can influence HIF-1 functioning and expression. It was revealed, that KLF2 promotes HIF-1α degradation in a von Hippel–Lindau protein-independent but proteasome-dependent manner. Finally, KLF2 disturbs the interaction between HIF-1α and HSP90 (its chaperone), which indicates that KLF2 promotes HIF-1α degradation by affecting its folding and maturation. Such findings identified KLF2 as a novel inhibitor of HIF-1α expression and function [58,68,69].

HIF is a subject of many investigations—its effect on many processes in organism has been studied. In the development of systemic and local inflammatory diseases, HIF may provide both pro-inflammatory and anti-inflammatory functions. Different isoforms of HIF can play different roles in the development of inflammatory and tumor diseases [1,4,5,70,71,72]. In particular, in colon cancer associated with a chronic colitis model, it was demonstrated that an increase in HIF-1 expression in intestinal epithelial cells does not lead to the formation of tumors or further cancer progression, but HIF-2-mediated inflammation contributes to the development of colon tumors, and HIF-2α activation in acute colitis causes severe inflammation [73,74].

### 2.2. HIF Expression Variability in Humans

Taking into consideration the role of HIF in hypoxia adaptation and the development of correlated hypoxia disorders, the differences in HIF (and its isoforms) basic expression level may determine the predisposition to the development of certain infectious, inflammatory and tumor processes, along with the already known factors such as age, gender, ethnicity, and others. People could be distinguished by significant individual variability in the expression levels of HIF and other HIF-dependent genes in leukocytes, which indicates phenotypic differences in its regulation. It was demonstrated that there is individual variability in the expression of HIF and its dependent genes in response to hypoxic or inflammatory stimuli, which, at least in part, is due to genetic polymorphisms [7,75,76,77,78,79].

A long-term study (over 8 years) of monozygotic twins at sea level and altitude demonstrated that ventilatory responses to hypoxic stimulus are rigid, a genetically determined physiological characteristic, reflecting the general non-specific reactivity of the organism [10]. In particular, it was revealed that the ability of human organisms to maintain relatively constant levels of oxygen consumption under hypoxic conditions (11% O_2_ 15 min) is mostly determined genetically (70–80%) and depends on individual sensitivity to hypoxia and hypercapnia.

In addition, the presence of numerous polymorphisms of the *HIF1A* gene and dependent genes indicates some genetic determinism of tolerance to hypoxia [16,80]. The presence of *HIF1A* gene polymorphisms in humans, which determines the high level of HIF-1α expression and functional activity, can contribute to growth in an aerobic capacity and the organism’s adaptation to hypoxia and physical activities [81,82,83,84]. For example, in humans, a missense polymorphism in the *HIF1A* gene, Pro582Ser, is presented in exon 12 (rs11549465 C/T), which increases HIF-1α protein stability and transcriptional activity [81]. Taking into account the role of HIF-1α in the regulation of gene expression involved in glycolytic reactions, muscle development and muscle tissue structures, it could be suggested that a functional *HIF1A* Pro582Ser polymorphism is associated with human physical performance. This fact was evidenced [85] after the detection of the 582Ser allele (Pro/Ser heterozygotes) ratio in Polish weightlifters, which was significantly higher than in the control group. Nevertheless, a basic HIF-1α high expression level, provided by polymorphisms, can facilitate worse prognosis in some diseases, including tumors [81,86,87,88,89]. Tibetan-specific allelic variations in the EPAS1 gene (*HIF-2A*), regulating the physiological reactions to high-altitude hypoxia through the HIF signaling pathway to maintain the hemoglobin levels of Tibetan highlanders at near-sea level values, were revealed [90]. The Sherpa-dominant EPAS1 haplotype may provide the fundamental genetic basis for hypoxia-tolerant oxygen sensing, resulting in Sherpas at high altitudes having efficient hemoglobin-oxygen transport systems [91].

The polymorphisms of prolyl hydroxylases PHD2 (EGLN1) and FIH-1 (HIF-1AN) were also studied. Several variants in EGLN1 5’-UTR were demonstrated to influence the sensitivity of acute mountain sickness (AMS) in the Chinese population [78]. Nonetheless, there was no correlation between any variants of HIF1A and VHL genes in Sherpas and the history of AMS [92]. In the work of [79] was demonstrated a correlation between single nucleotide polymorphisms in hypoxia-related genes such as PHD2 (SNPrs480902) and susceptibility to acute high-altitude pulmonary edema (HAPE). It was revealed that, in cases connected with HAPE, there was an extremely higher T-allele frequency than in the control group.

The concept of genetic contribution to the susceptibility to high-altitude diseases is supported by differences in susceptibility to hypoxia between populations, mostly provided by gene polymorphisms correlated with hypoxia adaptation [16,76,80,93,94,95]. Populations differ significantly according to the mechanisms of hypoxia tolerance. For instance, in contrast to the Andean highlanders, the Tibetans show a higher resting ventilation, hypoxic ventilatory response at the same altitude, higher nitric oxide (NO) levels in exhaled breath and blood, less hypoxic pulmonary vasoconstriction and lower pulmonary artery pressure, as well as a lower hemoglobin concentration and ratio of hemoglobin oxygen saturation [95]. A lot of studies at the moment try to explain and analyze the mechanisms of such differences.

Paying attention to the individual peculiarities in HIF and related gene expressions and the role of genetic factors in the mechanisms of high altitude-associated disorders may expand the boundaries of diagnostics and therapy of such diseases and substantiate mechanisms of hypoxia adaptation.

## 3. Physiological, Biochemical, and Molecular-Biological Characteristics of People with Different Tolerance to Hypoxia and Methods of Its Evaluation

Highlands, with their extreme natural conditions, primarily the lack of oxygen, are still relatively little developed and inhabitable. However, about 83 million people permanently live at altitudes above 2500 m—1.2 to 33% of highlanders suffer from chronic mountain sickness, and its prevalence depends on a number of factors, such as age, sex, altitude and ethnicity [95,96].

During climbing to an altitude of more than 2500–3000 m, the effect of severe acute hypobaric hypoxia is observed. It leads to the development of diseases such as AMS, HAPE and high-altitude cerebral edema (HACE), the sensitivity to which also differs [16,97,98,99,100,101].

### 3.1. AMS Susceptibility and Methods of Its Determination

AMS develops at altitudes above 2500 m, but in susceptible individuals it can be observed at lower altitudes. It is characterized by headache, nausea, fatigue, dizziness and insomnia, which usually occur within 6–12 h after a rapid ascent to an altitude of more than 2500 m in individuals not acclimatized to high altitudes [102]. AMS is less serious but has a much higher rate than two other acute altitude illnesses: HAPE and HACE. The AMS frames are between 40 and 90% with a reference to the altitude and individual susceptibility. For example, HACE is detected rarely at altitudes < 4000 m, and the possibility of it occurring between 4200 and 5500 m is estimated to be 0.5–1% [103]. Although the development of AMS has been extensively studied, its predominant pathogenesis mechanisms remain unclear.

The ability to adapt to oxygen deficiency quickly and fully is determined by the individual capabilities and basic tolerance of the organism. Individual tolerance to hypoxia depends on the intensity of oxygen consumption, metabolic characteristics, behavioral reactions, and a number of other individual differences. Understanding the mechanisms contributing the existence of differences in tolerance and adaptation to hypoxia is one of the main fields of research in high-altitude biology [104,105].

Except individual susceptibility, the group of main AMS determinants includes the altitude, the rate of ascent and the level of preacclimatisation. Moreover, various factors can also affect hypoxia susceptibility in mountainous conditions [16]. Different studies tried to reveal risk factors, which could be used for AMS susceptibility predictions. It was determined, that age [98], body mass index [106], arterial oxygen saturation [107], and sleep quality [108] have correlations with AMS susceptibility. Nonetheless, other studies revealed that these factors do not play important roles in AMS susceptibility [109]. In the work of [110] was shown that adults after the age 40–60 have a lower tendency to develop AMS than younger people. In contrast, a recent study demonstrated that there is no connection between the risk of developing AMS and age [111]. Therefore, searching for factors that are related to AMS development remains necessary.

Although sex steroidal hormones seem to influence ventilatory control [112], there is no clear evidence for gender differences in the acute responses and acclimatization to hypoxia [113]. Sex differences in respiration control and the incidence of respiratory infections have been revealed [114]. It was detected that repeated apnea and hypopnea are three times more likely to occur in men than in women [115]. However, according to [116], susceptibility to AMS does not differ according to gender. The authors of [117,118] found that women are more likely to develop AMS, but at the same time there are data demonstrating that, vice versa, men are more predisposed to suffer from AMS than women [119]. Thus, this question remains not fully understood and requires further research paying attention to the reproductive status and female estrous cycle phase.

In the study of [120] was stated that the occurrence of AMS is lower among smokers than among non-smokers. Moreover, recent evidence suggests that smoking may protect against AMS [121].

Thus, factors that determine susceptibility to high-altitude diseases are contradictory, but the indisputability of them is due to the individual tolerance to hypoxia. The inability to screen individual susceptibility to altitude diseases at sea level is a definite risk factor [16,122].

Clinical and pathophysiological studies, focused on the research on the effect of high-altitude conditions on the organism, made it possible to distinguish two polar groups of laboratory animals and humans according to their ability to adapt to the lack of oxygen and differences in their basic resistance to hypoxia [7,8,9,10,11,12,13,14,15,16,17,18,19,20,21,22]. In experimental studies, according to the results of the survival time (the time before respiratory disturbances and signs of asphyxia appear) in an assessment under conditions of hypobaric hypoxia, animals are divided into tolerant and susceptible to oxygen deficiency. Humans are mostly divided into tolerant and susceptible groups according to their AMS and HAPE sensitivity [72,73,74,75,76,77,78,79,80,81,82,83,84,85,86,87,88,89,90,91,92,93,94,95,96,97,98,99,100,101,123,124].

One of the most common methods for determining tolerance to oxygen deficiency is a model that reproduces the conditions of hypobaric hypoxia in decompression chambers, into which volunteers, pilots, astronauts or experimental animals are set [9,10,11,12,14,17,18,19,20,21,22,123,124,125,126,127,128,129]. To assess the individual’s hypoxia tolerance, people are exposed to altitudes of several thousand meters [99,101,130].

Human tolerance testing for AMS and HAPE is under development. A method for determining sensitivity to AMS using the Lake Louise symptom (LLS) score was suggested. This assessment is designed as a self-report questionnaire for research participants. Individuals demonstrating a cumulative LLS ≥ 3 with pronounced headaches after ascent to a high-altitude environment should be determined as susceptible. Another group with the sum of LLS ≤ 2 or not demonstrating headache after exposure to hypobaric hypoxia should be determined as tolerant [99,101,123,124,130,131]. Currently, LLS is a fairly objective method to assess the severity of AMS [131].

A hypoxia altitude simulation test (HAST) is known as a method of subject screening at risk of developing diseases associated with hypoxia (HAPE, AMS, etc.), when exposed to high-altitude or normobaric hypoxia. The HAST was first described by [132] for a preflight assessment of chronic obstructive pulmonary disease patients. During HAST, it is suggested that patients breathe the hypoxic air, corresponding to conditions equivalent of 2438 m above sea level during air travel. The hypoxic exposure duration is about 20 min, which provides a gas equilibrium, control of physiological reactions and assessment of possible symptoms. The exposure requires medical control and interpretation [133]. Although HAST was prescribed primarily for air travelers with respiratory diseases to assess the need for supplemental oxygen during air travel [133,134,135], it can be a useful tool for assessing tolerance to hypoxia also in patients with cardiovascular and other diseases, or even among healthy people [136,137]. For an AMS risk assessment, a similar test, using different degrees of hypoxia was suggested [136]. HAST can be performed in a hypobaric or normobaric hypoxic room, or simply by inhaling the hypoxic mixture through a mask. The main parameters studied are the values of the partial pressure of oxygen, oxygen saturation (SaO_2_), electrocardiography (ECG), blood pressure, blood lactate concentration, impaired lung function and the occurrence of medical symptoms. If people are expected to be physically active during a planned exposure to hypoxia, such as mountain climbing or work, HAST should be performed during exercise, such as in ergometry. The altitude dependence of the SaO_2_ value in AMS-susceptible and tolerant subjects was demonstrated; moreover, it is likely that SaO_2_ values during HAST are the most useful markers of susceptibility to AMS and HAPE [136,138]. 

In the work of [139] was suggested an evaluation model of individuals’ risk of altitude diseases (severe AMS, HACE or HAPE), but it supposes special breathing tests with a gas mixture, which makes it rather difficult for widespread use.

Furthermore, as additional indicators for determining the tolerance to hypoxia in humans, some biochemical parameters can be used—primarily, the evaluation of the lactate level in blood, which is used as a marker of inadequate tissue oxygenation [140]. However, Kushimoto et al. [141] noticed that lactate is not an absolutely reliable marker of tissue hypoxia, because hyperlactatemia can originate from aerobic glycolysis, which is not connected with tissue hypoxia [142]. Just as important is that the 2-nitroimidazole hypoxia marker, pimonidazole, was demonstrated to be effective as a marker of tissue hypoxia in both animal and human studies, especially in tumor research [143,144]. Nonetheless, all these methods suggest hypoxic exposure.

Since the problem of tolerance to O_2_ deficiency is relevant, an active search for individuals’ hypoxia and stress tolerance screening markers continues. The identification of specific molecules providing tolerance or susceptibility to stress factors, such as hypoxia, will play an important role in screening people for survival in adverse conditions, including spaceflight. Therefore, it could be advisable to search for molecular-biological markers, the concentration of which varies in individuals with different tolerances to oxygen deficiency under normoxia or under mild hypoxic exposure. However, there is a lack of markers described in the literature that would allow the evaluation of the tolerance to hypoxia in experimental animals and in humans without any exposure to oxygen deficiency.

In the investigation of [145] were defined three tests, which were possible physiological markers, reflecting the risk of AMS occurrence—heart rate variability, lung functions and cold pressor tests. Undoubtedly, at the moment sea level tests are not fully useful in predicting AMS; however, these indicators, to some extent, could be involved as references in forecasting susceptibility. Heart rate variability was investigated as a potential predictor for AMS in healthy organisms, while the main mechanism is still unclear [146]. Predicting AMS risk development before ascent may be relevant not only for highlanders but, what is more, for untrained individuals as improved transport technology allows the possibility of rapidly ascending to high altitudes.

According to [147,148,149], a marked increase in peripheral sympathetic activity is a common feature of mountain sickness. A higher manifestation of sympathetic activity is part of the integrated physiological response to hypoxic stimulus. Just as important is that subjects suffering from AMS had the abnormal profile of cardiovascular variability in comparison to subjects with no AMS detected [150]. Thus, the extent of sympathetic activity of the automatic nervous system assessment may be used to evaluate AMS susceptibility [151].

Subjects with AMS, furthermore, demonstrated a higher resting blood pressure at low altitudes. A connection between the resting blood pressure and the severity of AMS was revealed [150]. Pronounced studies are essential to find out whether and at which level of arterial blood pressure may indicate AMS development.

An indirect marker of low tolerance to AMS development may be increased anxiety at sea level [152]. It was demonstrated that trait anxiety at low altitudes was detected as an independent predictor of future pronounced AMS development at a high altitude. High-altitude state anxiety was independently associated with AMS and its severity.

Increased brain natriuretic peptide (BNP) levels definitely correlate with the severe AMS diagnosed using the LLS [153]. The BNP level is much greater in those with severe AMS at 5150 m. It was revealed that BNP levels correlate with the severity and prognosis of heart failure [154,155].

Genetic and proteomic studies demonstrated that particular biomarkers may be involved in the development of AMS. The levels of these biomarkers are different in individuals susceptible to AMS and people tolerant to AMS. In the work of [99] was found that molecules such as Insulin-like Growth Factor Binding Protein 6 (IGFBP6), Dickkopf WNT signaling pathway inhibitor 4 (Dkk4), Serum Amyloid A1 (SAA1), and Interleukin 17 Receptor A (IL-17RA) differ in AMS-susceptible and AMS-tolerant humans, and may serve as biomarkers of the sensitivity to AMS at low altitude [99].

In [101] was compared the changes in the proteome of blood plasma samples between the tolerant- and susceptible-to-AMS groups followed acute exposure to high altitudes. It was demonstrated that AMS-tolerant individuals are more capable of reducing oxygen consumption than the susceptible group after acute hypobaric hypoxic exposure. The level of proteins related to the tricarboxylic acid (TCA) cycle, glycolysis, ribosome, and proteasome after acute exposure to hypobaric hypoxia for 9 h at the altitude of 3800 m was significantly reduced in AMS-tolerant, but not in AMS-susceptible, groups. It was hypothesized that AMS-tolerant organisms after hypoxic exposure can reduce oxygen consumption by suppressing the TCA cycle and glycolysis, and reduce energy consumption by decreasing protein degradation and synthesis in comparison to AMS-susceptible individuals. In AMS-tolerant individuals, the inflammatory response may also be reduced as a result of the suppressed TCA cycle. It is supposed that the regulation possibility of oxygen consumption may play an important role in the development of AMS. However, further research is necessary to confirm this point. The proteomic method can be used to search for AMS-associated biomarkers via analyzing plasma samples from tolerant- and susceptible-to-AMS individuals. However, understanding of the specific AMS-related biomarkers is still insufficient.

In addition, in [123] was demonstrated that during exposure to a simulated altitude of 4875 m in a decompression chamber for 10 h, AMS-tolerant subjects had higher levels of Interleukin 1 Receptor Agonist, IL-1RA (after 4 and 9 h of exposure), and HSP70 (before exposure to hypoxia) in blood in comparison to AMS-susceptible subjects. Experimental evidence shows that HSPs, especially HSP70, can protect cells and organs against different types of damage. It is known that HSPs, in particular HSP70, prevent the degradation of HIF-1α by binding to its oxygen-dependent degradation domain (ODD) [156], therefore providing a protective effect under hypoxic conditions. HSP70 can probably serve as a potential marker of the organism’s high tolerance to oxygen deficiency at sea level. It was demonstrated that some polymorphisms of HSPs are responsible for a greater susceptibility to high-altitude diseases, and some less so. Reference [157] It was found out whether genetic variations in constitutive and inducible HSP70 genes provide association with the high-altitude illness risk [157]. Obtained data illustrate that individuals with HSP70-2 B/B and HSP70-hom A/B and B/B genotypes could be more susceptible to high-altitude illness, whereas those ones with HSP70-hom A/B genotype may be tolerant.

Macrophage Inflammatory Protein-1 (MIP-1) was higher in AMS-susceptible than in tolerant subjects after 4 h of hypoxia. Other studied biomarkers (IL-6, IL-8, IL-10, VEGF, TNFα, MCP-1, MMP-9) were not associated with AMS. Tolerance to AMS was accompanied by a pronounced anti-inflammatory response that could prevent subsequent pathophysiological events leading to AMS [123].

Thus, despite the large number of studies on the search for biomarkers of sensitivity to AMS, further research without hypoxic exposure is required.

### 3.2. HAPE Susceptibility and Methods of Its Determination

High-altitude pulmonary edema (HAPE) is a common condition among climbers. Approximately 10% of people develop HAPE within 24 h after a rapid ascent to 4500 m [158]. Although HAPE, if recognized early, can be easily prevented by a slow ascent and effectively treated with a rapid descent, it remains the most common reason of death associated with high altitudes. Therefore, it is necessary to develop methods for the prevention of HAPE by identifying susceptible people using simple non-invasive tests and providing them with appropriate recommendations for the rate of rise or prescription of preventive drugs [159]. Although the pathophysiological mechanisms of HAPE are not fully understood, it seems that an excessive increase in pressure in the pulmonary artery caused by hypoxia is a key factor, as evidenced by invasive and non-invasive measurements during HAPE [159,160,161,162].

People who had history of at least one episode of HAPE are considered susceptible. HAPE-tolerant individuals are considered the individuals who do not suffer HAPE over a 2-year high-altitude session, which includes 3 month staying at the extreme altitude > 4500 m. Invasive studies demonstrated that subjects susceptible to HAPE have an increased pulmonary vascular response during hypoxic exposure [163,164]. The increase in pressure in the pulmonary artery during exercises under normoxia was also more pronounced in HAPE-susceptible individuals than in tolerant people [164,165]. Using Doppler echocardiography, it was demonstrated that HAPE-susceptible subjects can have abnormal pulmonary vascular responses not only to hypoxia, but also to cycling on the back under normoxic conditions. Doppler echocardiography during exercise on a bicycle on the back or after 90 min of hypoxia can be a useful non-invasive screening technique for identifying subjects susceptible to HAPE [166].

As it was stated above, a rise in pulmonary artery pressure, induced by hypoxia, is a key factor in HAPE occurrence. According to [167], HAPE-susceptible subjects demonstrated both a baseline higher pulmonary artery pressure and reduced stroke volume in comparison with the control group, and higher baseline levels of BNP. Pronounced BNP levels also correlate with elevated pulmonary artery systolic pressure [168,169]. BNP levels could be easily measured and, therefore, may be an important marker for the HAPE susceptibility determination [167].

It was demonstrated that hypoxia exposure can negatively effect the NO production mediated by endothelial NO synthase (eNOS), and HAPE-susceptible people exhibit lower levels of exhaled NO compared to HAPE-tolerant subjects during acute exposure to hypoxia [170,171]. Impaired activity of eNOS and the loss of NO bioavailability were associated with endothelial cell dysfunction. This was the independent risk factor for cardiovascular diseases including atherosclerosis and hypertension [172,173]. Oxidative depletion of the eNOS cofactor tetrahydrobiopterin (BH4) can trigger eNOS uncoupling, in which the enzyme generates superoxide rather than NO [174,175,176,177]. eNOS uncoupling partially happens even in normal endothelium and may substantiate the predisposition of some individuals to endothelial dysfunction and cardiovascular complications [178].

In addition, it was observed that susceptible-to-HAPE people have a higher baseline serum HIF-1α level, as well as the concentration of T_3_ and atrial natriuretic peptide (ANP) [100]. Thus, the HIF-1α level during normoxia may be a representative and important marker for HAPE susceptibility determination. In spite of the important role of HIF-2 and HIF-3 in providing the response to hypoxic exposure mentioned above, the differences in their levels in people with different tolerances to high-altitude diseases remain unclear.

Thus, at present, individual differences in tolerance to hypoxia and high altitude-related diseases in humans were identified. They can be defined by a complex of factors, both genetic and phenotypic, and influence the predisposition to the development of certain disorders, including inflammatory and tumor diseases. Nevertheless, all existing methods for determining susceptibility to hypoxia and pathological high altitude-related conditions are carried out with the effect of oxygen deficiency of varying severity (Table 1). In addition, due to the different mechanisms of various high-altitude diseases, existing biomarkers may not be highly specific and universal. Thus, there are currently no standard markers for people differentiation according to hypoxia tolerance. At the same time, experimental studies are being carried out targeted at finding out the mechanisms of the formation of resistance to hypoxia and its consequences for the outcome of inflammatory and tumor diseases in laboratory animals.

## 4. Physiological, Biochemical, and Molecular-Biological Characteristics of Animals with Different Tolerances to Hypoxia and Methods of Its Evaluation

It was demonstrated that organisms with a highly organized central nervous system show more pronounced reactions to extreme influences, including hypoxia, in comparison to organisms with lower organization levels [179,180,181]. The human central nervous system in general, and, in particular, the cortex, are highly susceptible to O_2_ deficiency, while small mammals, such as rodents, and, especially, laboratory animals, have much higher density of capillaries in tissues, which contributes to their greater resistance to hypoxia [11,181]. In addition, invertebrates such as *Drosophila melanogaster*, *Caenorhabditis elegans*, *Daphnia magna* [182,183,184,185], and some vertebrates—individual fish species, naked mole rat, etc. [186,187,188]—are known as organisms tolerant to hypoxia. In many invertebrate and some ectothermic vertebrate species, hypometabolism maintenance also underlies the enormous tolerance to a big diversity of stress factors, including hypoxia, ischemia, and the hypothermia experienced in small mammalian hibernators [8]. Thus, methods for determining resistance to hypoxia in laboratory animals that are more tolerant than humans include the use of extreme altitudes.

### 4.1. Methods for Determining Hypoxia Tolerance in Animals

Experimental models of high-altitude human diseases mostly include the use of a decompression chamber [189,190,191]. Additionally, among experimental animals, the most common method for determining tolerance to oxygen deficiency is a model that reproduces the conditions of hypobaric hypoxia in decompression chambers by the controlled pumping out of air [11,14,18,19,128].

Usually for experimental animals, extreme altitudes, corresponding to respiratory disturbances and signs of asphyxia, are used. For outbred rats and Wistar rats, 11000–11500 m altitudes are used; for Sprague–Dawley rats, 9250–10668 m [9,11,12,14,18,19,126,192,193,194]. Another method for determining individual tolerance to hypoxia was proposed: a stepwise “ascent” of animals in a decompression chamber to platforms, corresponding to different altitudes until the recording of agonal breathing [195].

There is a method for determining resistance to hypoxia by breathing the gas mixture containing 3% oxygen in nitrogen, in the time from the beginning of inhalation to the onset of apnea. Inhalation of such a gas mixture is incompatible with life; however, the individual survival time of rats varies considerably—in different animals, respiratory arrest occurs within a period of 1 to 30 min [196].

At the current moment, there is no alternative method for organism selection into tolerant- and susceptible-to-hypoxia groups in experimental studies, except the determination of the survival time under conditions of acute hypoxic exposure in a decompression chamber or in conditions of breathing the gas mixture.

As a result of determining resistance to hypoxia, animals are divided into tolerant, normal and susceptible. The ratio may vary, depending on many factors (season, time of day, etc.). As practice shows, a significant ratio is accounted for normal animals (40–58%), the ratio of tolerant animals varies from 20 to 42%, and susceptible animals from 18 to 40% [9,11,12,126,194].

The determination of the hypoxia tolerance of animals, as a rule, is carried out once and the experiment is performed immediately after the exposure, after an hour, a week, two weeks, three weeks or a month [9,11,12,14,17,20,21,22,126,127,192,197,198,199]. Some authors carry out the test several times at some intervals; for example, three times with a one-week interval [11,12,14,127]. It is recommended to use an interval of one a month after determining the tolerance to hypoxia to eliminate the effect of hypoxic exposure and identify the initial differences between the phenotypes of animals [17,18,19,21,22]. It was demonstrated that, a month after the testing procedure in tolerant- and susceptible-to-hypoxia animals, the differences in many parameters remain [17,18,19,21,22,200]. However, it is not possible to find out whether these differences are a pre-existing feature or a result of hypoxic exposure without a method for determining tolerance to hypoxia excluding decompression chamber use. Therefore, it is advisable to search for molecular-biological markers, the concentration of which differs in organisms with different tolerances to hypoxia under conditions of normoxia or under compensated hypoxic exposure.

When determining the tolerance to hypoxia, it is necessary to take into account that it, as well as the tolerance to the development of infectious, inflammatory and tumor diseases, may depend on age and gender. In a number of experimental works, sex and age differences in the ability to adapt to high altitudes were revealed [201,202]. After hypoxic exposure, females recover their respiration faster than males [203]. In comparison to males, female rats are more tolerant to hypoxia: among female rats, tolerant-to-hypoxia organisms prevail, while males are predominantly susceptible and normal. We have identified sex differences in the morphological and functional state of the immune system, depending on hypoxia tolerance [200,204].

Newborn animals survive under an hour exposure to extremely pronounced degrees of hypoxia (13000 m). This could be possible due to the physiological immaturity of the nervous and endocrine systems, and the associated low oxygen consumption. We found that, in comparison to newborn and adult animals, prepubertal Wistar rats have the least tolerance to hypoxia, which correlates with the most pronounced manifestations of the systemic inflammatory response [204].

Other factors can also affect the accuracy of hypoxia tolerance measurement. Biological rhythms are important, but not fully understood, factors, influencing tolerance to hypoxia. According to the literature, the daily rhythm of the sensitivity of animals to the lack of O_2_ was established: in the evening and at night, the survival time under conditions of hypobaric hypoxia is less than in the daytime. It is also known that there are seasonal fluctuations in tolerance to hypoxia. The minimum of susceptible organisms was recorded in the autumn–winter period, and the maximum in summer. The ratio of tolerant-to-hypoxia animals was maximal from November to January and minimal in May–June [205,206]. In addition to daily and seasonal biorhythms, the infradian biorhythms of hormones and other processes were demonstrated [207]. We established the 4-day hypoxia tolerance infradian biorhythm in Wistar and Sprague–Dawley rats, which was in a phase with the biorhythm of corticosterone [193]. During the acrophase of the infradian 4-day biorhythm of corticosterone, the survival time of animals at altitude was higher than during its bathyphase, which was demonstrated both in Wistar and Sprague–Dawley rats, differing according to hypoxia tolerance. During the research of the sensitivity to O_2_ deficiency determination, it is necessary to take into account the existence of the 4-day fluctuations of this parameter [193].

Thus, the main method to determine tolerance to hypoxia in experimental animals is the use of a decompression chamber. Nevertheless, approaches used vary considerably depending on the study design and often do not take into account the influence of different factors. Thereafter, it is necessary to standardize the method for determining tolerance to hypoxia in experimental animal models.

### 4.2. Molecular-Biological, Pathophysiological and Biochemical Differences of Tolerant- and Susceptible-to-Hypoxia Animals

The studies based on using a decompression chamber made it possible to reveal differences in some parameters in tolerant- and susceptible-to-hypoxia animals; however, due to the variability of methods, the data are difficult to compare. It was discovered that tolerant- and susceptible-to-hypoxia animals differ in HIF-1 expression one month after the determination of tolerance to oxygen deficiency [13,18]. The feedback between the basic content of HIF-1 in the neocortex and the resistance of outbred male rats to hypoxia was detected: in susceptible organisms under normoxia, the level of HIF-1 was 1.7 times higher than in tolerant rats [13]. Thus, it was observed that tolerant- and susceptible-to-hypoxia animals differ in many parameters, including the expression of HIF-1. Therefore, normoxic HIF-1 level may be a representative and important marker for determination of hypoxia susceptibility. These findings are in accordance with studies on humans. As it was mentioned above, susceptible-to-HAPE people demonstrate higher serum HIF-1α levels during normoxia [100]. HIF-1 is considered a biomarker for the successful adaptation to various forms of hypoxia [35,208,209,210], and furthermore HIF-1 could be used as a selection diagnostic marker for individuals who have to work in extreme hypoxic conditions [211]. This question requires further attention. Moreover, despite the revealed differences in the expression of HIF-1 in tolerant- and susceptible-to-hypoxia animals, studies of other HIF isoforms (HIF-2 and HIF-3) have not yet been carried out in this context. Taking into account the role of HIFs in hypoxia adaptation and disease development, differences in the basic HIF expression level and its isoforms may determine a predisposition to the development of certain infectious, inflammatory, and tumor diseases, along with the already known age, sex, and other factors.

The structural differences of mitochondria, which play a pivotal role in oxygen sensing and free radical generation, in tolerant- and susceptible-to-hypoxia animals, studied one month after exposure to extreme simulated altitudes, were well-examined [17,21]. The authors demonstrated that the mitochondria of the cerebral cortex cells, liver and heart of tolerant- and susceptible-to-hypoxia rats differ in both structural and basic functional parameters. Under normoxia, a month after the determination of tolerance to hypoxia, cells of the cerebral cortex of tolerant rats were characterized by the high content of mitochondria with more densely packed cristae and dark matrix, large number of small mitochondria, and a higher concentration of mitochondrial enzymes such as Subunit A of Succinate Dehydrogenase (SDHA), Cytochrome b (Cyt b), Cytochrome C Oxidase Subunit I (COX1), and succinate versus mitochondria in susceptible rats. On the contrary, the number of mitochondrial cristae in the brain mitochondria of susceptible-to-hypoxia rats was less than in mitochondria in tolerant rats. Smaller mitochondria with a denser packing of cristae are functionally more active [15,212,213], which is consistent with the higher basic functional activity of mitochondrial energy apparatus in the cerebral cortex in tolerant-to-hypoxia rats in comparison to susceptible rats.

The large number of small mitochondria in the cerebral cortex in tolerant-to-hypoxia rats is an indicator of increased metabolic mitochondrial activity and higher intensity of oxidative phosphorylation in these rats in comparison to susceptible rats. This is in accordance with the results of earlier studies that demonstrated different intensities of oxidative phosphorylation in the cerebral cortex in rats with tolerant and susceptible hypoxia resistance [214,215]. Thus, under normoxia, phenotypic ultrastructural, functional and metabolic differences are observed between the mitochondria of cerebral cortex cells of tolerant- and susceptible-to-hypoxia rats. They indicate greater activity of the respiratory chain in rats tolerant-to-hypoxia in comparison to susceptible rats. These differences also suggest that energy metabolism is a determining factor in individual tolerance to hypoxia.

It was also observed that in liver cells mitochondria of tolerant-to-hypoxia animals, the rate of ATP-dependent K+ transport, which reflects the activity of the mitochondrial ATP-sensitive potassium channel, was higher. At the same time, the amount of K+ in the mitochondria of the liver and heart was higher in susceptible-to-hypoxia rats [9].

In the work of [22] was demonstrated that a month after the determination of tolerance to hypoxia, the rate of Ca^2+^ uptake by mitochondria of liver and heart cells in tolerant-to-hypoxia rats was higher than in susceptible rats, which is associated with differences in the level of some channel subunits in mitochondria. The ability to hold calcium in the mitochondria of liver cells in tolerant-to-hypoxia rats was 1.3 times higher than in susceptible rats. The results obtained indicate that in the liver and heart cells mitochondria in tolerant-to-hypoxia animals, functional and structural features in the transport of Ca^2+^ ions appear, which may be important for the functioning of mitochondria under hypoxic conditions, and contribute to the formation of adaptive traits that ensure the development of a cellular response to oxygen deficiency. These changes may underlie the sensitivity of cells to hypoxic damage at the molecular level.

The concept of the existence of different evolutionarily developed “functional and metabolic patterns” corresponding to two types of animals with different tolerances to acute oxygen deficiency has been proposed [9,15,21,216]. These patterns are based on the characteristic features of the energy apparatus, the state of the central nervous system and neurohumoral regulation, which determine the organism’s response to hypoxia. Tolerant-to-hypoxia animals are a type of organisms that differ from susceptible ones by maximally activated protective, antihypoxic mechanisms, which make them extremely resistant to short-term acute hypoxic effects. The specific ultrastructure of the mitochondria in tolerant and susceptible rats, demonstrated by [9,17,21,22], also supports the concept that animals with different tolerances to hypoxia have two different functional and metabolic profiles. They are associated with differences in the functional activity of the energy system, the status of membranes and receptor apparatus.

Animals belonging to two opposite types of hypoxia tolerance have significant differences in the effectiveness of energy support of the body, regulation of the central nervous and cardiovascular systems, neurohumoral regulation, stress-activating and stress-limiting systems, oxygen-transport function of blood and state of membranes and receptors [10,15,217].

Sympathetic regulation is predominant in susceptible rats while parasympathetic tone predominates in tolerant animals [218]. It is believed that susceptible-to-hypoxia animals have a weak type of nervous system, less developed internal inhibition, increased excitability and emotional reactivity. They respond to hypoxia with excitement and high locomotor activity. Susceptible-to-hypoxia animals are more prone to the development of diseases such as diabetes, obesity, thyrotoxicosis, atherosclerosis, etc. [15,217]. Such data are consistent with the information above about humans: indirect marker of susceptibility to AMS development may be increased anxiety at sea level [152]. On the contrary, in tolerant-to-hypoxia animals, excitability and anxiety are reduced, and moderate aggressiveness, more pronounced internal inhibition, low sensitivity to any provoking factors and a tendency to social domination are manifested, and they are more resistant to anesthesia. They react to acute hypoxia, cerebral ischemia, and carbon monoxide poisoning with an inhibitory reaction [15,217].

It was demonstrated that the physical endurance of the organism is significantly higher in tolerant-to-hypoxia rats than in susceptible rats, which indicates its direct relationship with tolerance to oxygen deficiency. It was observed that the lifespan of tolerant-to-hypoxia rats was 15% higher than that of susceptible rats [219].

In tolerant- and susceptible-to-extreme hypobaric hypoxia rats, some differences were found in the initial distribution of cardiac output: tolerant animals differed in less blood flow in skeletal muscles and had a higher blood flow in the brain, heart, kidneys, and lungs. These features are most pronounced between tolerant and susceptible rats during stress reactions. When determining tolerance by exposure to acute severe hypoxia (3% O_2_) in white outbred male rats, both tolerant and susceptible, there was a decrease in blood pressure; however, the degree of decrease was not the same—in tolerant animals with an initial mean blood pressure of 110–120 mm Hg there was a decrease to 50–60 mm Hg; in susceptible, to 30 mm Hg and below. The decrease in blood pressure in rats during hypoxia is explained by vasodilation in most organs and tissues, including kidneys, skeletal muscles, and brain. There is also a decrease in heart rate: in tolerant animals by 10–20 beats per minute, in susceptible animals, by more than 40–50 beats per minute. Consequently, tolerant-to-oxygen deficiency animals are characterized by a favorable oxygen regime and have a background for a longer survival under conditions of severe acute hypoxia in comparison with susceptible animals [196].

Research is also being conducted on various parameters of tolerant- and susceptible-to-hypoxia animals immediately after exposure to extreme simulated altitudes; in some cases, testing in a decompression chamber is performed three times at a certain interval. Predominantly, these works study the levels of activity of various enzymes, as well as the content of hormones in blood, since hypoxic exposure induces stress and contributes to a change in the concentration of catecholamines (epinephrine and norepinephrine) and corticosterone. Epinephrine and norepinephrine are released almost immediately after the sympathetic nervous system responds to stress. The content of norepinephrine immediately after the division of Sprague–Dawley rats into tolerant and susceptible at a simulated hypobaric hypoxia at 10668 m was investigated [126]. It turned out that the level of norepinephrine in blood plasma was significantly higher in tolerant-to-hypoxia animals than in susceptible. A significant increase in the norepinephrine concentration allows tolerant rats to cope with stress more efficiently than susceptible rats. The weight of the adrenal glands was significantly higher in tolerant-to-hypoxia rats than in susceptible ones [126].

The release of prolactin by the pituitary gland is considered a very sensitive marker of stress in mammals [220]. In addition to its participation in reproductive processes, prolactin plays a role in maintaining the equilibrium of the internal environment, regulating the functioning of the immune system, osmotic balance and angiogenesis. Adaptation to stress is associated with a diminished response of prolactin [220,221]. It was demonstrated that tolerant-to-hypoxia animals have significantly lower plasma prolactin levels than susceptible animals [126], which indicates a more effective adaptation of tolerant rats to hypoxic exposure.

According to [126], in the blood plasma of tolerant-to-hypoxia rats, immediately after a single measurement of tolerance to hypoxia, the content of Adrenocorticotropic hormone (ACTH) and testosterone was higher. It is known that in early stages after stress exposure the concentration of corticosterone in the blood plasma increases. The literature data on the content of corticosterone in tolerant- and susceptible-to-hypoxia rats are contradictory: according to [126], no significant differences were found in the level of total corticosterone in blood plasma in tolerant- and susceptible-to-hypoxia rats immediately after testing. However, according to [14], in the blood plasma of tolerant- and susceptible-to-hypoxia Sprague–Dawley rats immediately after a three-time hypoxic exposure at an extreme altitude, the corticosterone content was higher than in tolerant animals. Such differences are probably due to the variation in methods for determining hypoxia tolerance.

In the heart and blood plasma of Sprague–Dawley rats, immediately after three consecutive measurements of hypoxia tolerance, the level of erythropoietin and endothelin-1, which play an important role in maintaining vascular integrity, was studied. Endothelin regulates the tone of blood vessels in the lungs when exposed to hypoxic stress; in susceptible-to-hypoxia animals, its expression increased 30 times in the myocardium, which induces its hypertrophy and cardiac dysfunction [11,222]. In blood, its concentration was also increased in susceptible-to-hypoxia rats [14]. The content of erythropoietin, which stimulates erythropoiesis, increased in the heart and blood in tolerant-to-hypoxia rats, while in susceptible rats, on the contrary, it decreased [11,14].

In the heart of tolerant-to-hypoxia Sprague–Dawley rats, immediately after three consecutive measurements of hypoxia tolerance, the level of HIF-1α protein synthesis increased two times, while in susceptible rats the increase was not so significant [11]. We also showed an increase in the level of HIF-1 expression in tolerant-to-hypoxia Wistar rats after a single measurement of the resistance to oxygen deficiency, accompanied by an increase in VEGF and erythropoietin content [194]. An increase in the level of HIF-1 expression after hypoxic exposure in tolerant animals, apparently, contributes to their rapid and more effective acute adaptation to oxygen deficiency [208,210,223].

Nitric oxide (NO) is a potent vasodilator, which plays an anti-inflammatory role, shows antimicrobial action, antiplatelet activity, favors mitochondrial biogenesis (improved ATP production) and promotes angiogenesis [224,225,226]. The level of NO in the myocardium in tolerant-to-O_2_ deficiency animals increased two times, which increases the ability to maintain normal respiratory activity for a long time during acute hypoxia [11,14]. The activity of eNOS and iNOS in the myocardium was higher in tolerant-to-hypoxia rats than in susceptible rats [14]. As it was mentioned before, HAPE-susceptible people also show lower levels of exhaled NO in comparison to HAPE-tolerant subjects during acute exposure to hypoxia. It may be suggested that high NO levels could be used as biomarkers of pronounced hypoxia tolerance after acute hypoxic exposure.

In the plasma of tolerant-to-hypoxia animals, higher levels of NO were also detected in comparison to susceptible rats [127]. Increased levels of ROS and lipid peroxidation products reduce the availability of NO in circulation, which increases the risk of thrombosis [226]. Therefore, NO synthesis and its availability affect the final physiological result during hypoxia exposure. Due to the important role of NO in the blood pressure control, blood flow and other vital functions of the organism, it is extremely important to protect the available NO from the action of ROS during hypoxia. Reducing the production of oxidants or counteracting them by antioxidant systems during hypoxia can provide an increase in tolerance to hypoxia [227]. It was demonstrated that enzyme levels, such as superoxide dismutase and catalase, which protect cells from oxidative stress, is higher in the heart of tolerant-to-hypoxia Sprague–Dawley rats after a three-time hypoxic exposure [11]. Thus, the activity of these enzymes is increased in tolerant animals, which makes it possible to reduce the oxidative stress induced by hypoxia. At the same time, in susceptible-to-hypoxia animals, the activity of caspase-3, which promotes apoptosis, is higher [12]. The expression of the most studied heat-shock proteins—HSP27, HSP60, HSP70 and HSP90—in the myocardium of tolerant-to-hypoxia rats was significantly increased, which indicates their ability to overcome prolonged exposure to O_2_ deficiency and a more effective adaptation [11,14]. Differences in the level of HSP70 were found not only in rats with different tolerance to hypoxia, but also in people with different tolerances to AMS, as was mentioned above. Thus, HSP70 can be potential biomarker of a high tolerance to hypoxia.

Furthermore, studies of markers of oxidative stress were performed, and it turned out that the level of malondialdehyde, which is formed upon exposure to hypoxia, is almost eight times higher in the heart of susceptible-to-oxygen deficiency rats [11]. The level of carbonylated proteins characterizing oxidative stress increased in blood plasma in susceptible-to-hypoxia Sprague–Dawley rats immediately after the third hypoxic exposure at altitude, corresponding to 9754 m, which was more significant than in tolerant rats [127]. A low level of carbonylated proteins in blood plasma after three exposures to hypoxia at an extreme altitude with an interval of a week has been performed to be used as a marker of tolerance to hypoxia [127]. We also demonstrated that, 90 min after a single hypoxic exposure at extreme altitude, the level of the oxidative stress marker 8-isoprostane increased in the blood serum only in susceptible-to-hypoxia rats [194].

In the work of [228], a proteomic analysis of plasma proteins was conducted in rats exposed to hypoxia at 7620 m in a decompression chamber and 25 proteins were identified, whose expression changed during hypoxia. The majority of detected proteins were related with cellular defense mechanisms involving anti-inflammatory and antioxidant activities. Despite the fact that none of the proteins alone are definitely associated with hypobaric hypoxia, the combination of proteomic information of different proteins is significant in providing a better understanding of the molecular pathway affected by hypobaric hypoxia. Nonetheless, in this study, animals were not divided according to hypoxia tolerance. The extent of expression changes in the studied proteins under the influence of hypoxia could vary depending on the basic tolerance to oxygen deficiency. Any of these facts may be useful; however, the aim to find markers of hypoxia tolerance for clinical use and acclimatization remains relevant for research studies.

The available data on the characteristics of the reaction of the endocrine, antioxidant and other systems of organisms with different tolerances to hypoxia suggest the existence of differences in the response of other integrative systems, including the immune and closely related inflammatory processes in tolerant- and susceptible-to-oxygen deficiency animals.

Since it is known that hypoxia is associated with inflammation [1,2,4,46], the expression level of the nuclear factor NF-κB, which initiates inflammation, in the cytosol and nuclei cardiomyocytes in tolerant- and susceptible-to-hypoxia Sprague–Dawley rats was studied [14]. After a three-time exposure to simulated hypobaric hypoxia at 9250 m, the cytosolic expression of NF-κB decreased in the myocardium of susceptible-to-hypoxia rats and the nuclear expression increased, which indicates its activation. The expression of the proinflammatory cytokine TNFα in the myocardium of susceptible animals was higher, which also reflects the activation of the inflammatory response [14]. We have demonstrated the differences in the severity of the lipopolysaccharide (LPS)-induced inflammatory responses in male Wistar rats with different resistances to hypoxia. Rats susceptible to hypoxia are characterized by a more pronounced inflammatory response induced by LPS [18,19].

Obtained data on the content of interleukins, immunoglobulins and hormones in the peripheral blood of tolerant and susceptible-to-hypoxia animals are contrary [229,230,231]. According to [231], in tolerant-to-hypoxia rats, the serum IL-10 level was higher than in susceptible animals, and the concentration of IL-1β and TNFα did not differ; however, the period of the experiment after twice determining the tolerance to hypoxia was not indicated in the work. The level of immunoglobulins M, G and A also did not differ in tolerant- and susceptible-to-oxygen deficiency rats. The literature provides data about the absence of differences in the leukocyte formula in tolerant- and susceptible-to-hypoxia animals, as well as the cellular composition of the bone marrow and the level of lymphocytes in the thymus [230]. However, the work of [230] did not take into account gender differences; experiments were performed on males and females.

Since in experimental studies different lines of rats of both sexes are used, the extreme altitudes and the criteria for determining resistance to hypoxia vary significantly, the data presented in the literature are difficult to compare. Summary information on the differences between tolerant- and susceptible-to-hypoxia rats are presented in Table 2.

It is generally accepted to conduct experiments for one month after measuring tolerance to hypoxia, but this idea is not fully substantiated. It is not possible to find out whether the differences in some parameters revealed in tolerant- and susceptible-to-hypoxia animals are a pre-existing feature or a consequence of hypoxic exposure without a method for determining resistance to oxygen deficiency with no decompression chamber. In the future, it is necessary to conduct a further search for biomarkers of hypoxia resistance without exposure to altitude and to study the differences in the course of inflammatory and tumor diseases in organisms with different sensitivities to oxygen deficiency.

## 5. Conclusions

**Figure 1:**
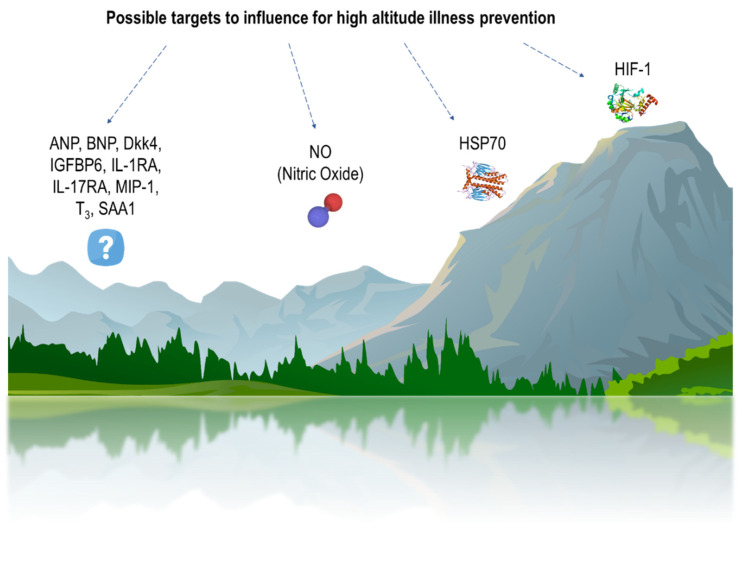
Common biomarkers, revealed both in humans and animals (HIF-1—Hypoxia-Inducible Factor, HSP70—Heat-Shock Protein 70, NO—Nitric Oxide), which are supposed to be responsible for determination of hypoxia tolerance and possible biomarkers, differences in which were demonstrated among humans with different AMS and HAPE tolerance (ANP—Atrial Natriuretic Peptide, BNP—Brain Natriuretic Peptide, Dkk4—Dickkopf WNT signaling pathway inhibitor 4, IGFBP6—Insulin-like Growth Factor Binding Protein 6, IL-1RA—Interleukin 1 Receptor Agonist, IL-17RA—Interleukin 17 Receptor A, MIP-1—Macrophage Inflammatory Protein-1, T_3_, SAA1—Serum Amyloid A1).

Thus, the existence of differences in the resistance of organisms to hypoxia and high-altitude diseases, depending on many factors, is clearly identified. Compelling results from various research groups suggested individual variability in hypoxia responses in humans and laboratory animals. Nevertheless, further research is needed to find out possible biomarkers of hypoxia tolerance, since the existing methods significantly vary, and the influence of external factors is not taken into account in studies. At the moment, several potential biomarkers can be identified to differentiate organisms by hypoxia tolerance, which were characterized in both experimental animals and humans. The main potential biomarkers are HIF-1, HSP70, and NO (Figure 1). HIF, apparently, plays a key role in the mechanisms of basic resistance to hypoxia. HIF is a subject of many investigations; however, individual differences in basic tolerance to hypoxia and HIF levels are not taken into account. Furthermore, differences in the content of some regulators of HIF (such as FIH, KLF2, etc.) and its isoforms in organisms with different tolerances to hypoxia remain uninvestigated. Moreover, the data about potential biomarkers were obtained after any hypoxic exposure. In addition, due to the different mechanisms of various high-altitude diseases, biomarkers may not be highly specific and universal. There is a continued search for the potential universal marker(s) for individual prognosis of reactions to hypoxia. Future investigations will be able to give an answer to this very important question. The identification of markers that make it possible to characterize hypoxia resistance without the effect of altitude will contribute to the development of personalized preventive medicine for various human diseases, especially mountain diseases, and could also be useful for people in the ascent of mountains, space exploration and other conditions associated with possible hypoxia.

## Figures and Tables

**Table 1:** Possible biomarkers of people tolerant and susceptible to acute mountain sickness (AMS) and high-altitude pulmonary edema (HAPE).

Disorder	Exposure	Tolerant	Susceptible	Ref.
AMS	Low altitude	Higher blood plasma level of IGFBP6	Higher blood plasma levels of Dkk4, IL-17RA, SAA1	[99]
	Low altitude and 3800 m for 3, 6, 9 and 12 h	Reduced level of proteins related to TCA, glycolysis, ribosome, and proteasome after 9 h		[101]
	Low altitude and 4875 m for 10 h	Higher blood level of IL-1RA (after 4 and 9 h of exposure), HSP70 (before exposure to hypoxia)	Higher blood level of MIP-1 after 4 h of hypoxia	[123]
	Low altitude and 4559 m		Higher resting blood pressure at low altitude	[150]
	Low altitude and over 9 consecutive altitudes during a progressive trek to 5140 m		Trait anxiety at low altitude was an independent predictor of future severe AMS development at high altitude	[152]
	At rest in 1300 m, following exercise and at rest at 4270 and 5150 m		BNP is significantly greater at 5150 m	[153]
HAPE	Normoxia		Higher baseline serum HIF-1α level, the plasma concentration of T_3_ and ANP	[100]
	Low altitude and at 4559 m		Exaggerated sympathetic activation	[149]
	Low altitude and 3100 m		Exaggerated pulmonary vascular response following ascent to high altitude	[163]
	Low altitude and at 3810 m		The increase in pulmonary artery pressure during exercises	[165]
	Low altitude and at 4500 m		Baseline higher levels of BNP, pulmonary artery pressure and reduced stroke volume	[167]
	Exposure to 4500 m		Lower levels of exhaled NO	[170,171]

**Table 2:** Total studied differences between tolerant- and susceptible-to-hypoxia animals, depending on the determination methods.

Targets	Methods	Tolerant	Susceptible	Ref.
Cerebral cortex cells	One month after a single measurement of tolerance to hypoxia	Higher content of mitochondria with more densely packed cristae and a dark matrix, number of small mitochondria, and concentration of SDHA, Cyt b, COX1, and succinate	Lower number of mitochondrial cristae	[17,21]
Liver cells		Higher rate of ATP-dependent K+ transport in the mitochondria Higher ability to hold Ca^2+^ in the mitochondria		[9,22]
Liver and heart cells		Higher rate of Ca^2+^ uptake by mitochondria	Higher amount of K+ in the mitochondria	[9,22]
Blood plasma	Immediately after a single measurement of tolerance to hypoxia	Higher levels of norepinephrine, ACTH, and testosterone	Higher prolactin level	[126]
	Immediately after three consecutive exposures at extreme altitude	Higher content of erythropoietin Higher level of NO	Higher levels of endothelin-1, corticosterone, ROS, and carbonylated proteins	[14,127]
Myocardium	Immediately after three consecutive exposures at extreme altitude	Higher levels of superoxide dismutase and catalase Higher levels of NO, erythropoietin and activity of eNOS and iNOS Higher expression of HIF-1α, GLUT1, HSP27, HSP60, HSP70, and HSP90	Higher activity of caspase-3, level of malondialdehyde, ROS, carbonylated proteins, expression of endothelin-1 and VEGF, the nuclear expression of NF-κB, and the expression of TNFα	[11,12,14]
Blood serum	5 min after a single hypoxic exposure at extreme altitude	Higher content of VEGF, erythropoietin, and TGF-β		[194]
	90 min after a single hypoxic exposure at extreme altitude		Higher content of TGF-β, level of the oxidative stress marker 8-isoprostane	[194]
	After double determining the tolerance to hypoxia	Higher serum IL-10 level		[231]
Liver and neocortex	5 min after a single hypoxic exposure at extreme altitude	Higher expression of HIF-1 and NF-kB		[194]
	One month after a single measurement of tolerance to hypoxia		Higher level of HIF-1	[13,18]
